# Clinical Cytogenetics of the Dog: A Review

**DOI:** 10.3390/ani11040947

**Published:** 2021-03-27

**Authors:** Izabela Szczerbal, Marek Switonski

**Affiliations:** Department of Genetics and Animal Breeding, Poznan University of Life Sciences, 60-637 Poznan, Poland; izabela.szczerbal@up.poznan.pl

**Keywords:** aneuploidy, cancer cytogenetics, centric fusion, chimerism, disorder of sex development, freemartinism, intersexualism, mosaicism, reciprocal translocation

## Abstract

**Simple Summary:**

The cytogenetic analysis of dogs is mainly focused on the diagnosis of disorders of sex development (DSD) and cancers. Unfortunately, the study of canine chromosomes is a challenging task due a high chromosome number (2n = 78) and the one-arm morphology of all autosomes. For years, the application of conventional cytogenetic techniques, Giemsa staining and G and DAPI (4′,6-diamidino-2-phenylindole) bandings, allowed the identification of sex chromosome aneuploidies and centric fusions. An advanced clinical cytogenetic diagnosis is also needed due to the fact that the dog is a valuable animal model in biomedical research. The application of hybridization methods, such as fluorescence in situ hybridization (FISH) and array comparative genome hybridization (aCGH), facilitated the detection of other chromosomal rearrangements. It can be foreseen that a wide use of modern molecular techniques (e.g., SNP microarray and next generation sequencing) will substantially extend the knowledge on canine chromosome mutations.

**Abstract:**

The dog is an important companion animal and has been recognized as a model in biomedical research. Its karyotype is characterized by a high chromosome number (2n = 78) and by the presence of one-arm autosomes, which are mostly small in size. This makes the dog a difficult subject for cytogenetic studies. However, there are some chromosome abnormalities that can be easily identified, such as sex chromosome aneuploidies, XX/XY leukocyte chimerism, and centric fusions (Robertsonian translocations). Fluorescence in situ hybridization (FISH) with the use of whole-chromosome painting or locus-specific probes has improved our ability to identify and characterize chromosomal abnormalities, including reciprocal translocations. The evaluation of sex chromosome complement is an important diagnostic step in dogs with disorders of sex development (DSD). In such cases, FISH can detect the copy number variants (CNVs) associated with the DSD phenotype. Since cancers are frequently diagnosed in dogs, cytogenetic evaluation of tumors has also been undertaken and specific chromosome mutations for some cancers have been reported. However, the study of meiotic, gamete, and embryo chromosomes is not very advanced. Knowledge of canine genome organization and new molecular tools, such as aCGH (array comparative genome hybridization), SNP (single nucleotide polymorphism) microarray, and ddPCR (droplet digital PCR) allow the identification of chromosomal rearrangements. It is anticipated that the comprehensive use of chromosome banding, FISH, and molecular techniques will substantially improve the diagnosis of chromosome abnormalities in dogs.

## 1. Introduction

The dog is the most important companion animal species, and one for which extreme interbreed phenotypic variation has arisen over the last 200 years [1]. One side effect of this intensive breeding, caused by genetic drift, is the preservation of undesired mutations in the gene pool. About 400 DNA variants that cause hereditary diseases in dogs have been described (Online Mendelian Inheritance in Animals—OMIA, https://omia.org/home/, (accessed on 20 December 2020)). 

Our knowledge of canine chromosomal mutations is less advanced, as the chromosome set of this species is a very difficult analytic subject. The diploid number of chromosomes is high (2n = 78), but the genome size of this species (2.4 Gb) is similar to other domestic mammals. The majority of chromosomes are thus small, and their banding patterns do not allow for unambiguous recognition of all homologs. Sex chromosomes are biarmed and easily recognizable, but all autosomes are acrocentric, and only the largest pair of chromosome 1 (CFA1, Canis Familiaris chromosome 1) is distinctly different from the other autosomes. 

There have been several attempts to arrange a reference banded karyotype of the dog (review by [2]). In 1993, the DogMap consortium, which was focused on the development of the canine marker genome map, suggested that a commonly accepted chromosome nomenclature for this species be established by a group of cytogeneticists experienced in canine chromosomes research. The international committee agreed that, due to the similarity of G-banding patterns of the small autosomes, only the largest 21 autosome pairs and the sex chromosome pair can be recognized with certainty. As a result of this work, a partial standard karyotype was developed [3]. An important step in characterizing the canine chromosomes was the use of a set of chromosome-specific painting probes for all autosomes and sex chromosomes [4]. Unfortunately, these probes are not available for diagnostic purposes. BAC (Bacterial Artificial Chromosome) probes with known chromosomal localization are very useful tools in clinical cytogenetics. Canine BAC clones can be purchased from the BAC libraries: CHORI-82 Canine boxer (Canis familiaris) BAC library and RPCI-81 Canine BAC Library (https://bacpacresources.org/clones.htm, accessed on 2 March 2021). Information on the localization of the CHORI-82 BAC clones is available in the CanFam3.1 assembly in NCBI - National Center for Biotechnology Information (Genome Data Viewer, accessed on 2 March 2021). Moreover, chromosome specific BAC clones from the RPCI-81 library were cytogenetically assigned [5,6]. Such probes can prove very helpful in recognizing all chromosomes, including the small autosomes (pairs 22–38) that are not included in the standard partial karyotype. However, it should be pointed out that conventional Giemsa staining is sufficient to identify sex chromosome aneuploidies and XX/XY leukocyte chimerism. Detecting centric fusions is also easy on Giemsa stained metaphase spreads, but identifying the autosomes involved requires the use of chromosome banding or fluorescence in situ hybridization (FISH).

The progress on various aspects of canine cytogenetics was reviewed by Breen [2] and by Reimann-Berg et al. [7]. In this article, we focus on the importance of chromosome analysis in diagnosing clinical cases. 

## 2. Sex Chromosome Aneuploidies

Although sex chromosome aneuploidies are an important cause of infertility and sterility, they have rarely been observed in dogs (Table 1). X monosomy has been reported in five female dogs to date, usually presenting abnormal estrus cycle and small ovaries, with no evidence of ovarian follicle development. Other abnormalities, such as small stature, juvenile appearance, or excessive skin in the ventrum of the neck (typical of Turner syndrome in women), were observed only in dogs with a pure monosomy, 77,X [8,9]. The vertical septum in the vagina, observed in a single case, could be coincidental [10]. The frequency of the monosomic cell line in individuals with mosaic karyotypes (77,X/78,XX) varied over a wide range, from 5% [10] to 95% [11]. This shows that a large number of metaphase spreads need to be evaluated in infertile female dogs suspected of X monosomy. 

The low incidence of X monosomy in female dogs is probably associated with the large size of the pseudoautosomal region (PAR), estimated at 6.4 Mb, which is more rich in genes than human or equine PAR [12,13]. The loss of one X chromosome is associated with the lack of a long PAR, leading to haploinsufficiency in a long genomic segment responsible for embryonic mortality. No correlation between PAR size and X trisomy was observed, which indicates that the overdose of PAR-located genes has no effect on the phenotype of X trisomy carriers [12].

Only a few females with X trisomy have been described in dogs, and they usually had abnormal estrous cycles and hypoplastic ovaries (Table 2). Interestingly, among the six reported cases with X trisomy, three females showed behavioral problems, such as fearfulness, lack of barking, or coprophagy. The majority of these cases had only a single cell line, 79,XXX. A mosaic 79,XXX/78,XX karyotype was incidentally diagnosed in a female dog with normal estrus [14]. It is worth mentioning that trisomic cell lines occurred in a low frequency (5%) of cells, which suggests that the frequency of the mosaic karyotype (79,XXX/78,XX)—associated with normal fertility or subfertility—may be underestimated.

The XXY complement has been found in six dogs, including two cases of XX/XXY mosaicism (Table 3). This abnormality is known as a cause of hyperplastic testes and sterility. However, other abnormalities, such as congenital heart disease and bilateral cryptorchidism, have also been described [19,20,21]. In some XXY dogs, testicular tumors were diagnosed [20,22] and the feminization of such dogs has also been reported [23].

## 3. Leukocyte Chimerism XX/XY

The XX/XY leukocyte chimerism, which is caused by the formation of anastomoses between the placentas of heterosexual fetuses, is associated with freemartinism, a form of disorder of sex development (DSD). The anastomoses enable the exchange of hematopoietic cells and molecules involved in sex differentiation between the fetuses [25]. The masculinizing factors (Sex Determining Region Y - SRY, which is a transcription factor; anti-Mullerian hormone and testosterone) produced by the fetal testes alter the sexual differentiation of the female fetus. This syndrome is well known in ruminants [26], but is also observed in other species, including dogs (Table 4). Between-species differences in the frequency of the chimerism are associated with the type of placenta: the high incidence of anastomoses in ruminants is associated with a cotyledonary organization of the placenta, while in carnivores the incidence is much lower due to the zonary organization of the placenta. It also seems that diffused placentas may be associated with an elevated risk of anastomoses in litters with a large number of fetuses, as has been observed in highly prolific lines of pigs [27]. This may suggest that in dogs, too, a greater number of puppies in a litter may be associated with a higher incidence of freemartinism.

The appearance of external genitalia is a major criterion for identifying DSD in dogs, but is less useful in freemartin dogs. Some freemartins present almost normal female genitalia [28], while others have a normal male appearance [29,30]; in sporadic cases there are ambiguous external genitalia [31].

It is important to point out that virilization can be caused by freemartinism or by testicular or ovotesticular XX DSD; a correct diagnosis should thus be made using cytogenetic analysis. A comprehensive study of six French bulldogs with ambiguous external genitalia revealed that five were testicular or ovotesticular XX DSD, while one was a freemartin [32]. Using the nomenclature of DSD dogs, XX/XY leukocyte chimerism can be considered testicular, ovarian, or ovotesticular DSD [33]. These forms are also observed in dogs and, as in ruminants, there is no correlation between the percentage of XY cells and the extent of the virilization. In the reported cases, the proportion of the XY cell line ranged from 15% to 80% (Table 4).

**Table 4:** Leukocyte chimerism XX/XY reported in dogs.

Proportion of XX and XY Cell Lines [%]	Breed	Phenotypic Sex Considered by Owners	Characteristic Feature of Phenotype	Reference
Lack of information	Schipperke	female	Enlarged clitoris, testis and ovotestis, uterus,	[34]
43/57	Pug	female	Enlarged clitoris, hypospadias, no signs of estrus, testis and ovotestis	[23]
45/55	Dachshund	male	Abnormal urogenital tract, hematuria, ovaries	[35]
Lack of information	Spaniel × Papillon	unknown	Ovaries	[36]
Lack of information	American Eskimo	female	Normal vulva and clitoris, ovotestis	[37]
Lack of information	Spaniel	unknown	Small penis, empty rudimentary scrotum, uterus, ovaries with reduced number of follicles	[38]
85/15	Belgium Shepherd	male	Aggressive behavior, intersexuality, abdominal testes, underdeveloped penis, urethra ended under the anus, vas deferens connected to an oviduct, blind uterus	[29]
Lack of information	Fila Brasileiro	male	Prepuce-like structure located closer to the anus, testicles with an immature epididymides	[39]
43/57	Border Terrier	male	Undeveloped penis, ovarian-like structure	[30]
70/30	Wirehaired Pointing Griffon	female	Primary anestrus, juvenile vulva, enlarged clitoris, testis	[28]
78/22	Shih Tzu	ambiguous	Residual penis with a prepuce located in a position typical of a male, prostate, gonads remained undetectable	[31]
20/80	French Bulldog	female	Enlarge clitoris, ovotestes	[32]
30/70	Great Dane	female	Underdeveloped internal reproductive organs, rudimentary testicles	[40]
54/46	Great Dane	female	Undeveloped foreskin	[14]

The diagnosis of XX/XY leukocyte chimerism also requires the analysis of another tissue, such as hair follicles or buccal epithelial cells, in order to distinguish between leukocyte chimerism and whole-body chimerism. Moreover, it facilitates the establishment of concordance between phenotypic and chromosomal sex. Since external genitalia of freemartins are often ambiguous, it cannot be excluded that some cases are incorrectly considered by owners as males. In Table 4, two cases were described as males, but this was not confirmed by cytogenetic or molecular studies of other tissue.

**Figure 1:**
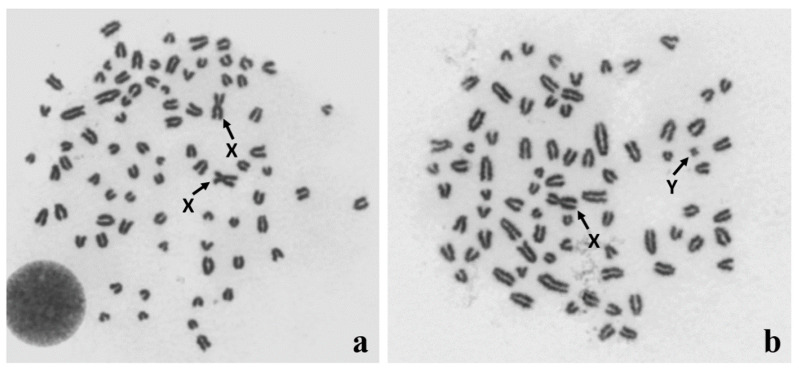
Identification of (**a**) 78,XX and (**b**) 78,XY Giemsa-stained metaphase spreads from in vitro cultured leukocytes obtained from a DSD (disorder of sex development) dog. Sex chromosomes are indicated with arrows.

A common diagnostic strategy involves the cytogenetic analysis of leukocytes (Figure 1), the molecular detection of Y-linked genes (e.g., *SRY* and *ZFY*), or microsatellite markers in DNA samples derived from the blood and the second tissue [31]. Droplet digital PCR (ddPCR) has recently been demonstrated to be a fast and reliable method for detecting XX/XY leukocyte chimerism in cattle and pigs [27,41]. This method can also be recommended for DSD diagnosis in dogs.

## 4. Structural Chromosome Rearrangements

**Figure 2:**
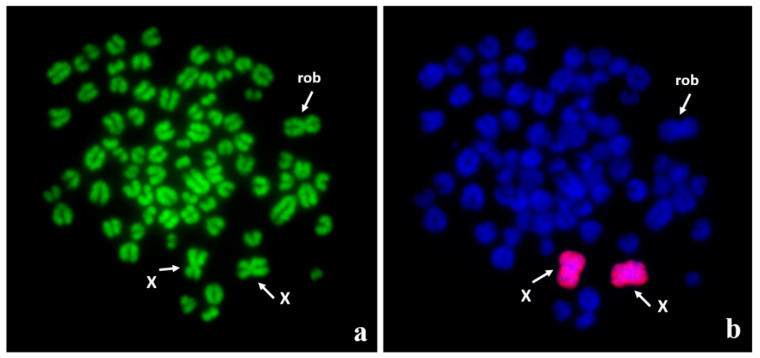
Robertsonian translocation, 77,XX, rob(5;23) in an infertile DSD female dog: (**a**) Q-banded metaphase spread with three biarmed chromosomes; (**b**) the same metaphase spread after fluorescence in situ hybridization (FISH) using whole X chromosome painting probe to facilitate recognition of X chromosomes from the fused chromosome (rob). The autosomes involved in the translocation were identified by FISH with locus-specific probes (for details, see [42]).

Robertsonian translocations (centric fusions) have been reported quite often in dogs, probably due to their ease of identification. Such mutations lead to a reduction in the diploid chromosome number to 77 and the formation of a biarmed derivative (der) chromosome (Figure 2). The first Robertsonian translocations in dogs were described in the 1960s, and over a dozen different translocations have now been reported in total (Table 5). Different chromosomes are involved in these mutations, but in several cases no attempt was undertaken to indicate the autosomes involved. Two autosomes, named CFA13 and CFA23 (Canis Familiaris chromosome 13 and 23), were identified more often, but this finding should be taken with caution due to the difficulties in identifying small autosomes when only chromosome banding was used. There is only a single report on the use of FISH with locus-specific probes to describe a centric fusion, which found rob(5;23), as described by Switonski et al. [42].

Dogs with Robertsonian translocations present normal phenotype, but there is often a small decrease in fertility. However, this abnormality has also been diagnosed in infertile bitches [43,44,45], which either showed a lack of estrus or were unsuccessfully mated many times. Moreover, two dogs with persistent Müllerian duct syndrome (PMDS) were found to have centric fusions, but coincidentally [46,47]. An interesting case of centric fusion in a testicular/ovotesticular XX DSD (*SRY*-negative) female dog with an enlarged clitoris and uterus was described by Switonski et al. [42]. CFA5 and CFA23 were involved in this rearrangement and the fusion led to a pericentromeric fragment of CFA23 being deleted. It was hypothesized that this could cause the deletion of regulatory sequences for genes that are important in ovarian development located in CFA23, such as *PISRT1*, *FOXL2*, and *CTNNB1*.

**Table 5:** Robertsonian translocations reported in dogs.

Chromosome Involved in the Fusion	Breed (Number of Cases)	Characteristic Feature of Phenotype	Reference
Not identified	Mixed terrier (1)	Cardiac defect	[48]
Not identified	Miniature Poodle (1)	Bone chondrodysplasia	[49]
Not identified	Setter–Retriever cross (1)	Phenotypically and clinically normal female	[50]
13 and 17	Golden Retriever cross (1)	Normal, fertile female	[51]
13 and 23	Golden Retriever-type (1 + 11 offspring)	Normal phenotype with the exception of congenital inguinal hernia in two female homozygotes in progeny	[52]
1 and 31	Poodle (6, including 1 homozygote male)	Normal phenotype	[53]
21 and 33	Walker Hound (1 + sister and 4 offspring)	Narrow vulva, absence of estrus	[43]
Not identified	Mixed breed (1)	Infertile female	[44]
8 and 14	West Highland White Terrier (1)	Infertile female, normal reproductive organs	[45]
5 and 23	Bernese Mountain Dog (1)	XX DSD, *SRY*-negative enlarged clitoris, testicle, ovotestis, uterus	[42]
Not identified	Miniature Schnauzer (1)	XY DSD, PMDS (Persistent Mullerian Duct Syndrome)	[46]
1 and unidentified	Miniature Schnauzer (1)	XY DSD, PMDS	[47]
Not identified	American Staffordshire Terrier (1)	XX DSD, *SRY*-negative (Sex Determining Region Y) enlarged clitoris, ovotestis,	[14]

Reciprocal translocations have rarely been diagnosed in dogs, probably due to difficulties in the recognition of one-armed autosomes. Until now, only three X/autosome mutations have been found, and this was possible because the translocation chromosome derived from the X had the abnormal morphology. The first mutation was identified in a male-to-female sex-reversed Yorkshire terrier [54]. The dog had two cell lines—a normal 78,XY and a line with X-autosome translocation. The mutation was identified using a whole X-chromosome painting probe which showed the hybridization signals on the X chromosome and unidentified autosome. Recently, two new cases of such rearrangement were observed in two female dogs with abnormalities of the genitourinary system [14]. In one of the female dogs, a pure X/autosome translocation, 78,X,t(X;2), was found, while in the second case a mosaicism of 78,X,t(X;A)/78,XX was observed. The cell line with the translocation occurred with a low incidence and it was not possible to identify the autosome involved.

It can be foreseen that the detection of canine chromosome translocations could be more efficient if a more sophisticated tool, such as multihybridization slides with a set of canine subtelomeric probes, were available, as has been recently developed for the chromosomes of pigs [55,56] and cattle [57].

## 5. Cytogenetic Characterization of Other Forms of DSD Cases

**Figure 3:**
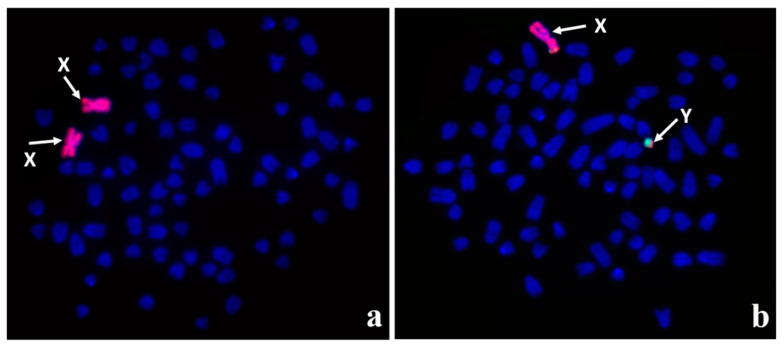
Identification of sex chromosomes by FISH with the use of painting probes (X: red) and (Y: green): (**a**) 78,XX, (**b**) 78,XY, with visible signals in pseudoautosomal region (PAR).

Cytogenetic analysis is a crucial step in classifying DSD [33,58]. Some DSD dogs may have chromosome abnormalities (sex chromosome DSD), as described above, but the majority of cases have a normal chromosome set described as XX DSD or XY DSD. Sex chromosomes are usually identified in such cases by Giemsa staining, karyotyping of R-banded chromosomes, or FISH with chromosome-specific probes (Figure 3) [32,59,60,61].

**Figure 4:**
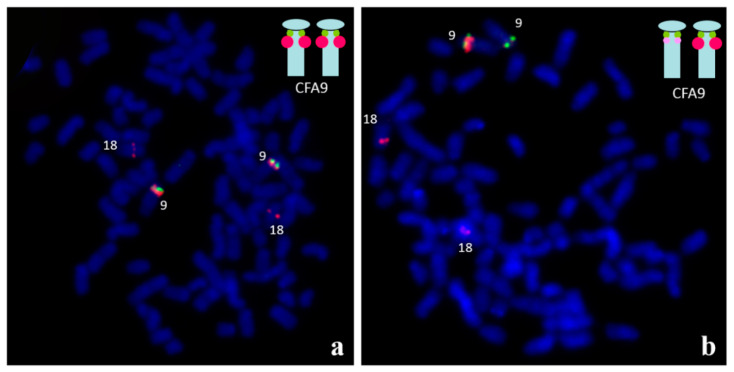
Identification of the copy number variation (CNV) in the region of *SOX9*, located on CFA9. Two BAC (Bacterial Artificial Chromosome) clones were used: the green signals are specific to the *SOX9* gene and the red signals are specific to the upstream CNV. The red probe also presents homology to CFA18. (**a**) The two large red signals on CFA9 indicate multiplication of the CNV region. (**b**) Another example of the variation - the single large, red signal is visible on one CFA9 chromosome, only.

Cytogenetic analysis is also helpful for visualizing copy number variation (CNV). It has been shown that, in some dogs, CNVs in the *SOX9* gene region (CFA9) are associated with XX DSD phenotype. FISH with BAC probes specific to this region was used to identify duplication or multiplication (Figure 4) [62,63,64].

**Figure 5:**
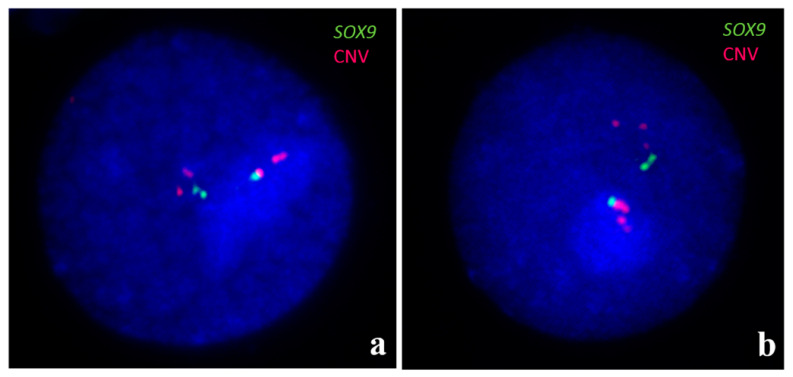
FISH for identification of three copies of *SOX9* gene (green signals) in two interphase nuclei (**a**,**b**). Moreover, multiple copies at CNV region upstream *SOX9*, as well as homologous region of CFA18 (red signals), are visible. For details, see [64].

Since the resolution of the hybridization signals on metaphase chromosomes is not sufficient to detect *SOX9* gene triplication, interphase nuclei were examined (Figure 5) [64]. It should be mentioned that molecular techniques such as MLPA (Multiplex Ligation-dependent Probe Amplification) and a-CGH (array Comparative Genome Hybridization) have also been employed for the identification of this CNV [60,61,62].

## 6. Sperm Cytogenetics

The dog is a valuable large animal model in studies of human reproduction and development [65]. The segregation of sperm into two fractions, rich in, respectively, X-chromosome or Y-chromosome bearing cells, is an assisted reproductive biotechnology that has been developed for dogs. The effectiveness of this technology can be validated using the FISH technique with molecular probes specific to sex chromosomes on the segregated sperm samples. Dual color FISH has shown that the sorting of dog sperm by flow cytometry is very efficient, and the purity of the sorted samples was high at about 90% [66]. Komaki et al. [67] also used FISH to evaluate sex chromosome aneuploidy in sperm. The authors performed three color FISH with probes for chromosomes CFAX, CFAY and CFA1. Altogether, the sperms from eight dogs were analyzed and the mean frequencies of aneuploidy were: 0.016% (XX), 0.024% (YY), 0.08% (XY), and 0.176% (lack of sex chromosomes but with CFA1).

There has to date been no information on chromosome abnormalities in oocytes or embryos, despite the fact that studies of in vitro embryo production in dogs are quite advanced [68]. It can be foreseen that more such studies will be undertaken due to the increase in interest in biomedical research using induced pluripotent somatic cells [69].

## 7. Cancer Cytogenetics

Cancer is a genetic disease caused by gene or chromosomal mutations, classified as germline (inherited) or somatic. The somatic mutations can cause the disease or can have an effect on its development. Knowledge of the germline mutations responsible for cancer development in domestic animals, including dogs, is scarce [70]. Studies to identify mutations in cancer cells are also poorly advanced in domestic animals, though canine cancers have been considered more frequently than others [71].

Cytogenetic studies of canine cancer have a long history, with the first papers being published almost sixty years ago [72]. Early reports of chromosome abnormalities in canine tumors should be taken with caution due to the difficulties in recognizing autosomes. Since the partial international standard karyotype of the dog was agreed on in 1996, and since chromosome-specific molecular probes for FISH became available in the late 1990s, we focused this review on reports published after 1996.

Sex chromosome and autosomal aneuploidies, as well as centric fusions, are easy to identify as it was already mentioned earlier; however, the identification of small autosomes involved in such abnormalities is challenging. There are several reports showing a clonal predominance of specific aneuploidies in canine cancer cells. Analysis of G-banded chromosomes of cells derived from thyroid adenomas showed that the trisomy of chromosome 18 (CFA18) was predominant in the studied metaphase spreads [73]. Another study of in vitro cultured lymphocytes derived from the bone marrow of two dogs suffering from acute myeloid leukemia revealed two clonal aberrations: a trisomy of chromosome 1 (CFA1) and a chromosome translocation t(X;8) [74]. These aberrations were identified using G-banding. Polysomy of chromosome 13 (CFA13), caused by centric fusion between these chromosomes, was observed with an elevated frequency in cells derived from the prostate carcinomas of two dogs [75,76]. Interestingly, aberrations of this chromosome have also been observed in other dog cancers [7].

The introduction of molecular techniques into chromosome analysis was a very important step for canine cancer cytogenetics. Establishing the canine BAC library allowed the identification of BAC clones harboring 25 candidate genes for different cancers, which could be used in FISH analysis of cancer cells [77]. Researchers have searched for BAC clones in the canine genome library to use as FISH probes. Using this approach, it was shown by Breen and Modiano [78] that the well-known somatic chromosome rearrangements associated with some human cancers are also present in canine counterparts. These researchers examined the canine counterparts of three human cancers: chronic myelogenous leukemia (CML) associated with *BCR* and *ABL* fusion, sporadic Burkitt lymphoma (BL) associated with *MYC-IgH* fusion, and chronic lymphocytic leukemia/small lymphocytic lymphoma (CLL) associated with a hemizygous deletion harboring the *RB1* gene. They found that approximately 25% of the metaphase spreads or interphase nuclei of cancer cells they studied carried similar chromosome rearrangements. This study confirmed that the dog is a valuable animal model for studies of human cancerogenesis. The colocalization of *BCR-ABL* was also detected by FISH in dogs suffering from chronic monocytic leukemia (CMoL) [79] and acute myeloblastic leukemia without maturation (AML-M1) [80]. Canine BAC clones and whole chromosome painting probes were used by Vozdova et al. [81], who studied canine cutaneous mast cell tumors. Among different clonal aneuploidies and structural rearrangements, the most common was trisomy of CFA11.

Complex chromosome rearrangements causing genomic imbalances (loss or gain of genetic material) can be efficiently analyzed by comparative genomic hybridization (CGH). However, the classic CGH approach requires the reliable identification of banded chromosomes. The first attempt to use CGH to analyze canine cancer cells was by Dunn et al. [82], who studied a glial tumor cell line. Unfortunately, difficulties with chromosome recognition meant it was not possible to present a detailed characterization of the imbalances. The study showed that the only abnormality observed in all metaphase spreads was CFA1 trisomy. To overcome problems with identifying chromosomes using banding techniques, a molecular version of the CGH, called array CGH (aCGH), was developed. The first canine aCGH for 87 canine BAC clones was presented by Thomas et al. [83]. Soon after, two advanced aCGH microarrays were developed. One included 1158 canine BAC clones harboring canine genome fragments distributed along all chromosomes, with an average interval of 2 Mb [84]. In the second, the distribution of the BAC clones was approximately 10 Mb [85]. These molecular tools have replaced classical cytogenetic techniques in studies of complex chromosome rearrangements in cancer cells.

## 8. Conclusions and Perspectives

Although great progress has been achieved in studies of the organization of the canine genome, analysis of its chromosomes remains challenging. It is not surprising that the majority of abnormalities identified so far are sex chromosome abnormalities and centric fusions, as these can be identified by conventional Giemsa staining. The identification of sex chromosomes in DSD dogs plays a very important role in elucidating the DSD background, so classical analysis of chromosome preparations should be a common diagnostic approach. It facilitates the identification of sex chromosome abnormalities (e.g., X monosomy and XXY trisomy) or of abnormal sets of sex chromosomes in leukocytes (XX/XY leukocyte chimerism). The unequivocal identification of structural chromosome rearrangements in which small autosomes are involved usually requires the use of the FISH technique with probes derived from the canine BAC library. A promising perspective is related with the application of synthetic oligonucleotide probes (oligos) designed with the use of computational tools. The oligonucleotide libraries can be a valuable source of probes specific for a given chromosome, its region or a single gene [86,87,88].

It can also be expected that, in the near future, molecular techniques will play an important role in animal clinical cytogenetics. One of such techniques is digital droplet PCR (ddPCR), which allows the determination of the number of X and Y chromosome copies and the detection of sex chromosome aneuploidies and XX/XY leukocyte chimerism in a rapid, reliable manner. Other molecular techniques such as arrayCGH, SNP-microarray, MLPA, and NGS (next generation sequencing) are already very useful in human clinical cytogenetics in detecting structural rearrangements [89,90]. Moreover, the application of BioNano technologies offers the detection of chromosomal abnormalities, CNVs and structural variants [89] with a high resolution [91]. A very recent update of the canine genome reference sequence [92] should facilitate the successful use of the sequencing technologies. Taken together, it is expected that the spectrum of traditional cytogenetic techniques used in clinical diagnosis will be replaced by advanced DNA-based technologies, which are named “cytogenomics” [93].

The development of canine cytogenetics/cytogenomics also depends on the interest of veterinarians and dog breeders, who should be aware of the importance of such testing. Since the dog is an important biomedical animal, it may be expected that new diagnostic tools will be developed to overcome the difficulties of chromosome identification.

## Figures and Tables

**Table 1:** Cases of X monosomy reported in dogs.

Karyotype	No. of Cells Analyzed	Breed	Characteristic Feature of Phenotype	Reference
77,X	Lack of information	Doberman Pinscher	Small stature, excessive skin in the ventrum of the neck, no signs of estrus, small ovaries consisting primarily of interstitial-type cells and solid epithelial cords	[8]
77,X	60	Miniature American Eskimo	Juvenile appearance, signs of proestrus, small and fibrous ovaries, no evidence of ovarian follicle development or corpora lutea	[9]
77,X[95%]/78,XX[5%]	40	Toy Poodle	Abnormal estrus cycle and apparently persistent follicles, gonadal dysgenesis	[11]
77,X[5%]/78,XX[95%]	220	Munsterlander	Infertility, vertical septum in vagina	[10]
77,X[6%]/78,XX[94%]	473	Bearded Collie	Infertility, irregular and poorly manifested estrus cycles	[10]

**Table 2:** Cases of X trisomy reported in dogs.

Karyotype	Breed	Characteristic Feature of Phenotype	Reference
79,XXX	Airedale Terrier	Primary anestrus, ovaries with solid epithelial cords and large masses of interstitial cells, lack of follicles and corpora lutea	[15]
79,XXX	Mixed breed	Infertility, normal reproductive organs, ovaries with primary follicles and corpora lutea, dental anomalies, abnormal behavior (lack of barking and fearfulness)	[16]
79,XXX	Labrador Retriever	Primary anestrus, chronic dermatitis, abnormal behavior (coprophagy)	[17]
79,XXX	Silky Terrier	Infertility, abnormal estrous cycles, hypoplastic ovaries, absence of normal follicular structures, shy and timid behavior	[18]
79,XXX	Labrador Retriever	Infertility, abnormal estrous cycles, hypoplastic ovaries, absence of normal follicular structures	[18]
79,XXX/78,XX	Boston Terrier	Estrus symptoms occurred once, ovary with corpora lutea	[14]

**Table 3:** Cases of the XXY complement reported in dogs.

Karyotype	Breed	Characteristic Feature of Phenotype	Reference
79,XXY	German Shorthair Pointer	Testicular hypoplasia, lack of spermatogenesis, ventricular septal defect, congenital heart abnormalities	[19]
79,XXY	Great Dane	Female external and internal genitalia, structure reminiscent of a vestigial scrotal sac	[23]
79,XXY	Norwich Terrier	Testicular dysgenesis, azoospermia	[24]
79,XXY	West Highland White Terrier	High stature, rugae of the dermis and hypodermis, low level of testosterone, Sertoli cell tumor	[22]
79,XXY/78,XY	Miniature Schnauzer	Alopecia, gynecomastia, bilateral cryptorchidism, Sertoli cell tumor	[20]
79,XXY[18%]/78,XY[82%]	Poodle	Bilateral cryptorchidism, testes with vacuolation of the seminal cells and small nests of Leydig cells, total absence of sperm cells	[21]

## Data Availability

Data sharing is not applicable to this article as no new data were created or analyzed in this study.
